# Antibiotic stewardship in the intensive care unit

**DOI:** 10.1186/s13054-014-0480-6

**Published:** 2014-08-13

**Authors:** Charles-Edouard Luyt, Nicolas Bréchot, Jean-Louis Trouillet, Jean Chastre

**Affiliations:** Medical-Surgical Intensive Care Unit, ICAN, Institute of Cardiometabolism and Nutrition, Hôpital de la Pitié-Salpêtrière, Assistance Publique-Hôpitaux de Paris, Université Pierre et Marie Curie, Paris 6, 47, bd de l’Hôpital, 75651 Paris, Cedex 13 France

## Abstract

The rapid emergence and dissemination of antimicrobial-resistant microorganisms in ICUs worldwide constitute a problem of crisis dimensions. The root causes of this problem are multifactorial, but the core issues are clear. The emergence of antibiotic resistance is highly correlated with selective pressure resulting from inappropriate use of these drugs. Appropriate antibiotic stewardship in ICUs includes not only rapid identification and optimal treatment of bacterial infections in these critically ill patients, based on pharmacokinetic-pharmacodynamic characteristics, but also improving our ability to avoid administering unnecessary broad-spectrum antibiotics, shortening the duration of their administration, and reducing the numbers of patients receiving undue antibiotic therapy. Either we will be able to implement such a policy or we and our patients will face an uncontrollable surge of very difficult-to-treat pathogens.

## Introduction

Optimal antibiotic use is crucial in the critical care setting, especially in an era of rising antibiotic resistance and lack of new antimicrobial development [1-3]. Study results indicate that 30% to 60% of antibiotics prescribed in ICUs are unnecessary, inappropriate, or suboptimal [4-7]. Overprescribing and misprescribing antibiotics are undoubtedly contributing to the growing challenges posed by antibiotic-resistant bacteria, and epidemiological studies have clearly demonstrated direct relationships between antibiotic consumption and the emergence and dissemination of resistant strains in hospitals and ICUs [7-20]. As defined by the Society of Healthcare Epidemiology of America and Infectious Diseases Society of America (IDSA) Joint Committee on the Prevention of Antimicrobial Resistance in hospitals, ‘stewardship of antimicrobials is an apt descriptor of related activities that help optimize antimicrobial therapy, ensuring the best clinical outcome for the patient while lowering the risk of subsequent development of antimicrobial resistance’ [14]. Thus, in-ICU antibiotic stewardship encompasses rapid identification of patients with bacterial infections, better empirical treatment selection, using pharmacokinetic-pharmacodynamic (PK-PD) characteristics to optimize antibiotic dosing and administration modalities, de-escalation once culture results become available, shortening therapy duration, and reducing the numbers of patients treated unnecessarily.

Unfortunately, improving in-ICU antibiotic use is particularly difficult for three main reasons: infection severity often precludes withdrawing or postponing antibiotics, the complex decision-making process frequently involves doctors with limited expertise, and it is difficult to ensure disease-long continuity of care by the same medical team 24 hours a day, 7 days a week. Here, we review how in-ICU antibiotic therapy could possibly be optimized and rationalized.

## Rapid identification of intensive care unit patients with bacterial infections

Most published observational data suggest that the time to appropriate antibiotic administration is a major outcome determinant for ICU patients with severe bacterial infections. Indeed, each hour of delay in administering effective antibiotics for septic shock is associated with measurably increased mortality [6,21-25]. Thus, as strongly recommended by all guidelines [26-29], obtaining biological specimens should not postpone timely antibiotic administration to patients with severe sepsis or septic shock.

However, owing to methodological concerns, the harmful effects of inadequate therapy are not accepted by all [30-36]. Because in-ICU signs and symptoms of infection due to non-infectious causes are common, rushing to prescribe antibiotics may mean that many uninfected patients receive unnecessary treatment. In a quasi-experimental, before-and-after, observational cohort study of patients admitted to the University of Virginia surgical ICU, Hranjec and colleagues [32] postulated that delaying antibiotics for hemodynamically stable patients with suspected infections (35% pneumonia) until they were objectively documented would not worsen mortality. Notably, that conservative approach was associated with lower all-cause mortality, more initially appropriate therapy, and shorter mean treatment duration than the aggressive strategy. Thus, for clinically stable patients, that strategy might achieve better antibiotic use without impacting prognosis. Obtaining specimens for appropriate cultures before antibiotic administration is essential to confirm infection, identify responsible pathogens, and enable therapy de-escalation in response to susceptibility profiles.

The inaccuracy of conventional approaches to diagnose hospital-acquired infections (HAIs) and the impossibility of those strategies to avoid antibiotic overprescription led some investigators to hypothesize that using biological markers - for example, C-reactive protein, soluble-triggering receptor expressed on myeloid cells-1, or procalcitonin (PCT) - might better identify true bacterial infections and facilitate therapeutic decisions. However, although PCT is a good marker of community-acquired infections (CAIs), it does not seem to be for HAIs [37-41]. Indeed, blood PCT concentrations can rise in various non-septic conditions: major trauma, surgery, acute respiratory distress syndrome, multiorgan failure, post-transplantation rejection, cardiogenic shock, severe burns, heat stroke, and so on. Thus, high PCT concentrations the day sepsis is suspected are non-contributory because increases that are attributable to a prior non-infectious condition or active infection cannot be distinguished [39,42,43]. Moreover, PCT can remain low in some microbiologically proven bacterial infections, either because the infection remains contained in a tissue compartment that can synthesize PCT locally without systemic release, thereby explaining the low serum level despite true infection, or because of a 24- to 48-hour lag time in infection onset to peak PCT release. Thus, intensivists are rightly reluctant to rely exclusively on biological markers when severe infection is suspected [37,38,43-47].

## Selection of initial antibiotic therapy

Owing to the emergence of multiresistant Gram-negative bacilli (GNB) (for example, *Pseudomonas aeruginosa*, extended-spectrum β-lactamase-producing Enterobacteriaceae, and carbapenemase-producing *Klebsiella pneumoniae*) and the increasing role of Gram-positive bacteria (like methicillin-resistant *Staphylococcus aureus*, or MRSA), empirical broad-spectrum antibiotics are justified for most ICU patients with clinically suspected HAIs [25-27,48]. Regimen choice should be based on local antimicrobial susceptibility patterns and anticipated side effects while considering the antibiotics received within the preceding 2 weeks and striving whenever possible not to use the same classes [49-51]. Having current and frequently updated knowledge of local bacteriological epidemiology increases the likelihood of prescribing appropriate initial antibiotics. Whether surveillance cultures could further improve empirical treatment selection for ICU patients with suspected hospital-acquired pneumonia (HAP) is still debated but certainly should be weighed when difficult-to-treat microorganisms abound, making initial choices particularly risky [52,53]. Observational study results confirmed that initial regimens combining a broad-spectrum β-lactam and an aminoglycoside increased the proportion of appropriately treated patients compared with monotherapy or a combination of β-lactam and fluoroquinolone [54,55]. Only patients with mildly or moderately severe, early-onset infections and no specific risk factors (for example, prolonged hospitalization, immunosuppression, or recent prolonged antibiotics or a combination of these) can receive a relatively narrow-spectrum drug, like a non-pseudomonal third-generation cephalosporin.

For ICU patients admitted with health care-associated or community-onset infections or CAIs, more restraints for antimicrobial therapy selection are certainly possible. For example, it is increasingly recognized that applying current criteria for health care-associated pneumonia - hospitalization for at least 2 days during the preceding 90 days, residence in a nursing home or extended-care facility, home intravenous (antibiotics or chemotherapy) therapy, and chronic dialysis or home wound care (or both) during the preceding 30 days - as indications for broad-spectrum antibiotics may lead to overtreatment of many patients with pneumonia [56-62]. To address this conceptual limitation, investigators developed multiple risk-assessment models that refine those criteria [61,63,64]. Available data suggest that the incidence of pathogens resistant to the usual in-patient IDSA-American Thoracic Society guideline-recommended antibiotic regimen (that is, a non-pseudomonal cephalosporin and a macrolide) is usually not significantly increased unless two or more risk factors are present, with prior antibiotic use or hospitalization and poor functional status being more important predictors of resistant bacteria than nursing-home residence alone [61]. Using such an algorithm could lead to fewer pneumonia patients unnecessarily receiving broad-spectrum antibiotics.

Within the past decade, the way clinical microbiology laboratories identify microorganisms was revolutionized, leaving behind slow traditional methods based on phenotype characteristics (for example, growth on defined media, colony morphology, Gram staining, and biochemical reactions) incurring significant diagnosis delay, in exchange for new diagnostic techniques (real-time multiplex polymerase chain reaction and matrix-assisted laser desorption/ionization time-of-flight mass spectrometry) [65,66]. The latter, making possible rapid pathogen identification and their antimicrobial resistance patterns (at least for certain organisms), could undoubtedly promote earlier therapy appropriateness and de-escalation [67]. Multiple instrument platforms, marketed by well-established manufacturers, are beginning to displace or complement (or both) automated conventional phenotyping tools, providing accurate microbial identification from blood cultures within 1 to 2 hours. Nevertheless, it is unlikely that any of those new diagnostic methods will completely replace phenotyping for antibiotic susceptibility testing in the near future.

Pending the complete development of those above-mentioned techniques, Bouza and colleagues [68] described simple microbiology laboratory-accessible, rapid, antimicrobial susceptibility E-tests directly on samples (lower respiratory tract or other biological specimens) to improve early appropriate in-ICU antimicrobial choices. In a prospective randomized study of 250 patients with microbiologically confirmed ventilator-associated pneumonia (VAP), the authors showed that reporting rapid E-test-obtained antibiotic susceptibility of responsible microorganisms to the treating physicians (mean ± standard deviation: 1.4 ± 0.75 days post-sampling versus 4.2 days with standard methods) was associated with fewer days of fever and antibiotics until VAP-episode resolution, less antibiotic consumption, less *Clostridium difficile*-associated diarrhea, lower antimicrobial costs, and fewer days on mechanical ventilation (MV) [68].

## Pharmacokinetic-pharmacodynamic-optimized antimicrobial therapy

Reported findings demonstrated the need to individually adjust the antibiotic target doses and administration modalities to treat severe bacterial infection to each patient’s PK and putative or documented pathogens’ susceptibilities, as assessed by their minimal inhibitory concentrations (MICs) [69-73]. Most investigators distinguish antimicrobials by their killing mechanism: concentration-dependent (for example, aminoglycosides and fluoroquinolones) or time-dependent (for example, β-lactams and carbapenem). The most important PK-PD parameters are peak concentration/MIC >8-10 and 24-hour area under the concentration curve (AUC)/MIC >100-125 for aminoglycosides and fluoroquinolones. For β-lactams and carbapenem, the blood concentration should be maintained for >90-100% of the between-dose interval above MIC, at least in the case of severe infection [74,75]. However, it should be acknowledged that the exact target for PK-PD-optimized therapy remains elusive. Some antibiotics, such as fluoroquinolones and glycopeptides, are more complex and exhibit both concentration- and time-dependent kill characteristics where the best predictor of efficacy is the AUC/MIC. Others, such as carbapenems, have a marked post-antibiotic effect (that is, lead to a prolonged suppression of bacterial growth even with antibiotic concentrations below the MIC) [76,77].

**Figure 1:**
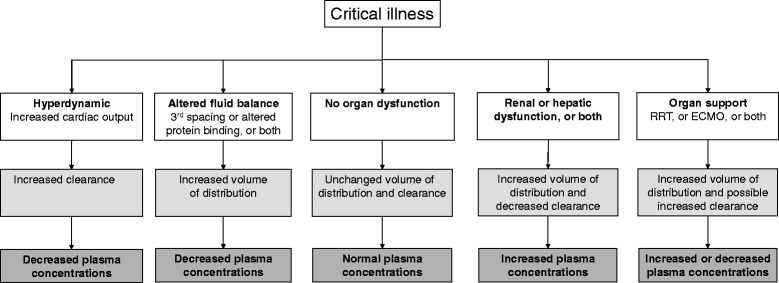
**Pathophysiological changes commonly observed in critically ill patients and their effects on drug concentrations.** Reproduced with permission from Elsevier Limited [75]. ECMO, extracorporeal membrane oxygenation; RRT, renal replacement therapy.

**Figure 2:**
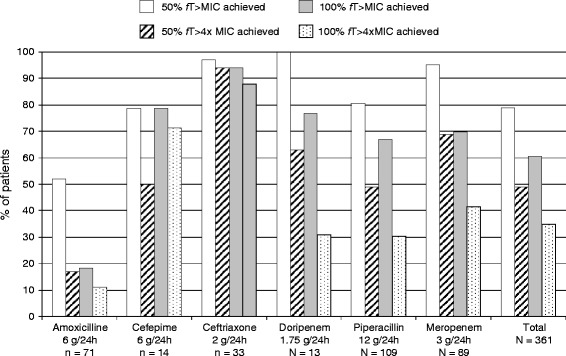
**Achievement of pharmacokinetic/pharmacodynamic targets in intensive care unit patients according to antibiotics used.** Data are expressed as percentage of patients achieving target. Doses for each antibiotic are given as a median. This figure was drawn from data in Table 3 of [5] with permission from Oxford Journals. 50% *f*T > MIC, free drug concentration maintained above minimum inhibitory concentration of the known or suspected pathogen for at least 50% of dosing interval; 50% *f*T > 4 × MIC, free drug concentration maintained above a concentration fourfold higher than the minimum inhibitory concentration of the known or suspected pathogen for at least 50% of dosing interval; 100% *f*T > MIC, free drug concentration maintained above minimum inhibitory concentration of the known or suspected pathogen throughout the entire dosing interval; 100% *f*T > 4 × MIC, free drug concentration maintained above a concentration fourfold higher than the minimum inhibitory concentration of the known or suspected pathogen throughout the entire dosing interval.

ICU patients’ altered PK secondary to increased volume of distribution and decreased elimination can result in insufficient serum aminoglycosides or β-lactam concentrations (or both) when standard doses are administered, emphasizing the need to carefully monitor peak and trough antibiotic levels when treating resistant pathogens, respectively [5,78,79]. Antibiotic doses for ICU patients derived from other patient groups are likely to be suboptimal because of significant antibiotic PK changes, particularly volume of distribution and clearance. Organ support techniques, including renal replacement therapy and extracorporeal membrane oxygenation, increase PK variability (Figure 1) [80-82]. In a recent prospective study conducted at 64 hospitals worldwide, 20% and 40% of 248 ICU patients receiving β-lactams for infection did not achieve free antibiotic concentrations above their pathogens’ MICs during 50% and 100% (50% and 100% *f*T > MIC, respectively) of the dosing interval (Figure 2) [5]. Frequently, higher than usually recommended antibiotic doses or continuous or extended infusions (or a combination of these) are needed [5,70,71,73,79,83-85]. Interestingly, use of prolonged infusion appeared to be associated with a significant reduction in mortality and improvement in clinical success when compared with intermittent boluses in a recent meta-analysis of 29 studies (18 randomized controlled trials and 11 observational studies) with a total of 2,206 patients [85].

Development of *a priori* dosing algorithms based on MIC, creatinine clearance and weight, and the clinician-specified AUC target might improve management of these patients, obtaining more precise antibiotic use than current guidelines [73,79,83,84,86]. Ultimately, adjusting antibiotic doses based on pathogen MICs and daily free antibiotic blood concentrations may reach optimized PK-PD targets in most ICU patients. A therapeutic drug-monitoring strategy, compared with traditional dosing methods, might not only reduce clinical failure rates in ICU patients but also prevent adverse events due to too high (toxic) antibiotic levels [87,88].

A double-blind randomized trial comparing 7 days of doripenem three times a day (4-hour infusion of 1 gram) with 10 days of imipenem-cilastin (1-hour infusion of 1 gram) for GNB VAP was prematurely stopped after random assignment of 274 patients because of inferior efficacy and higher day-28 all-cause mortality in the subgroup of doripenem-treated, *P. aeruginosa*-infected patients [89]. Despite longer doripenem infusions to optimize targeted antibiotic concentrations above the pathogens’ MICs during the 8-hour interval, this protocol performed more poorly, clearly documenting the risk associated with a so-called PK-PD-optimized antibiotic strategy when blood concentrations cannot be monitored and adjusted to stay above the responsible pathogens’ MICs. Perhaps the treatment duration or concentrations (or both) were sub-therapeutic for patients with elevated creatinine clearance, clearly documenting the risk associated with a so-called PK-PD-optimized antibiotic strategy when blood concentrations cannot be monitored and adjusted to stay above the MIC of the responsible pathogens [90].

For patients on MV, aerosolized antibiotics delivered via an efficient system, synchronized with inspiration, achieved airway drug concentrations 100- to 300-fold higher than the MICs of most bacteria, including multidrug-resistant pathogens [91-95]. Those levels, without systemic toxicity, might eradicate proximal airway pathogens in patients on MV and lower the pressure for selection of new resistant organisms, as shown in a recent, double-blind, placebo-controlled study of 42 ICU patients who required prolonged MV and who were colonized or infected (or both) with potentially difficult-to-treat pathogens (for example, MRSA and non-fermenting GNB) [96]. However, larger clinical trials must confirm those findings before that strategy can be recommended, in light of its potentially deleterious impact on the local epidemiology when used for all ICU patients over prolonged periods [97-99].

## Antimicrobial therapy de-escalation

The need to ensure that ICU patients with true bacterial infections receive prompt and appropriate antibiotics can lead to many more patients receiving antimicrobials than necessary, because of non-specific clinical signs of infection. This is particularly true for HAP/VAP, which represents the first in-ICU indication for starting antibiotics. Thus, regardless of the diagnostic strategy used for suspected HAP/VAP, serial clinical and microbiological evaluations are highly relevant to re-assess therapy after 48 to 72 hours and to stop it if infection is unlikely [48,100]. To accomplish that goal, each ICU should design its own diagnostic decision-tree strategy to manage patients with clinically suspected HAP/VAP to identify those with a low probability of infection, whose therapy can be discontinued when infection appears improbable [27,48]. At least, antibiotics should be withdrawn when the following three criteria are fulfilled on day 3: (a) the clinical diagnosis of pneumonia is unlikely - no definite infiltrates seen on repeat chest radiography and only one of the following three findings is present: temperature greater than 38.3°C, leukocytosis (greater than 12,000/mm^3^) or leukopenia (less than 4,000/mm^3^), or purulent tracheobronchial secretions - or an alternative non-infectious diagnosis is confirmed; (b) non-significant tracheobronchial aspirate culture results; and (c) no severe sepsis or shock [101]. Direct examination of distal pulmonary samples collected by bronchoalveolar lavage with or without a bronchoscope and quantitative culture results have consistently yielded fewer microorganisms growing above the diagnostic threshold than qualitative tracheal aspirate cultures [48,102]. Pertinently, when therapeutic decisions were based on those results, compared with the clinical approach, fewer patients received antibiotics that were of a potentially narrower spectrum, thereby limiting the emergence and dissemination of drug-resistant strains and minimizing antibiotic-related toxicity [103].

For many ICU patients with infections (including late-onset infections), therapy can be de-escalated, once respiratory tract, blood, or other specimen culture results become available, if no resistant organism (for example, *P. aeruginosa*, *Acinetobacter* spp., or MRSA) is recovered or because the isolated pathogen is sensitive to a narrower-spectrum antibiotic than that prescribed empirically [26,27,48]. For example, if MRSA is not found, vancomycin and linezolid should be stopped unless the patient is allergic to β-lactams or has developed an infection with Gram-positive bacteria susceptible only to them. Very-broad-spectrum agents (like carbapenems, piperacillin-tazobactam, and cefepime) should also be restricted to patients whose infectious pathogens are susceptible only to them. Because fluoroquinolones have been associated with the emergence of resistant strains, their in-ICU use probably should be discouraged [104,105]. Antifungals should never be prescribed for *Candida* isolated from respiratory secretions alone [106]. However, clinicians should know that, when third-generation cephalosporins are chosen to treat infections caused by *Enterobacteriacaea* with inducible β-lacatamase (*Enterobacter*, *Citrobacter*, *Morganella morganii*, indole-positive *Proteus*, or *Serratia* spp.), the emergence of resistant variants may lead to treatment failure. Unfortunately, study results showed that de-escalation, though not associated with any adverse outcomes, was not consistently applied in many ICUs [107-111].

The two most commonly cited reasons to prescribe combined antibiotics for the entire treatment duration are to achieve synergy and to prevent the emergence of resistant strains. However, antibiotic synergy has been shown to be valuable only *in vitro* and in patients with neutropenia, bacteremia, or a greater than 25% probability of death [25,112-122]. Randomized controlled trial results on combined therapy showed its benefit to be inconsistent or null, even when they were pooled in meta-analyses or when analysis was restricted to *P. aeruginosa*-infected patients [113,121,123,124]. Importantly, such regimens did not prevent the emergence of antimicrobial resistance during therapy and were associated with significantly more nephrotoxicity [121]. Those observations were confirmed in a randomized, open-label trial on 600 patients meeting criteria for severe sepsis or septic shock: combined meropenem and moxifloxacin versus meropenem alone did not achieve less organ failure or better survival or any secondary endpoints [113]. Based on those data, most patients’ therapy could be safely switched to monotherapy after 3 to 5 days, provided that the initial therapy was appropriate, the clinical course evolved favorably, and microbiological data did not indicate difficult-to-treat microorganisms, with high *in vitro* MICs, as can be observed for some non-fermenting GNB.

## Shortening treatment duration

Although shortening the duration of antibiotic administration for ICU patients may represent the most powerful strategy to reduce antibiotic impact on resistance emergence, most guidelines still recommend relatively prolonged or imprecise durations [26,28,125,126]. Efforts to shorten the duration for bacterial infections are justified by study results on the natural history of therapeutic responses. Most patients who had CAIs or HAIs, including VAP, and who received appropriate antimicrobial therapy had good clinical responses within the first 6 days [127-129]. Prolonged therapy facilitates colonization with antibiotic-resistant bacteria, which may precede recurrent infectious episodes.

Results of a multicenter, randomized controlled trial on 401 patients with microbiologically proven VAP showed that their clinical outcomes were similar to those of patients receiving appropriate empirical therapy for 8 or 15 days [130]. Relapse rates for short-duration therapy tended to be higher when *P. aeruginosa* or *Acinetobacter* spp. was the causative agent, but clinical outcomes were indistinguishable. Those observations were confirmed by trials that evaluated an antibiotic discontinuation policy for patients with other infections [111,131-138].

**Figure 3:**
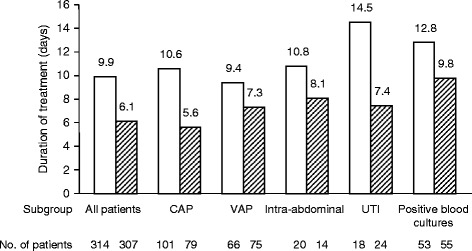
**Duration of antibiotic treatment of the first episode in the PRORATA trial, according to infection site.** White bars indicate patients included in the control group. Hatched bars indicate patients included in the procalcitonin-guided group. This figure was drawn from data in Table 2 of [37] with permission from Elsevier Limited. CAP, community-acquired pneumonia; PRORATA, Use of Procalcitonin to Reduce Patients’ Exposure to Antibiotics in Intensive Care Units; UTI, urinary tract infection; VAP, ventilator-associated pneumonia.

Many clinicians remain reluctant to prescribe fewer days of antibiotics for patients with severe HAIs and prefer tailoring antibiotic duration to the ensuing clinical course or using serial biomarker (for example, PCT) determinations (or both). The rationale for using the latter to customize treatment duration relies on evidence that the inflammatory response is often proportional to infection severity. When the response is absent or mild, antibiotics might logically be discontinued earlier. Thus, adapting treatment duration to PCT kinetics seems reasonable and was demonstrated to be useful in several randomized trials, including seven in the ICU, targeting patients with acute respiratory infections [37,41,139-143]. The largest of those studies was the PRORATA trial that included 621 ICU patients; 67% of these patients were on MV, 50% had CAIs, and 50% had HAIs, and half of them had septic shock [37]; patients in the PCT group had significantly more (mean ± standard deviation) days without antibiotics than controls (14.3 ± 9.1 versus 11.6 ± 8.2; absolute difference 2.7 days; 95% confidence interval 1.4 to 4.1; *P* < 0.0001), and this lower antibiotic consumption was not associated with poorer outcomes. Furthermore, regardless of infection site and the infectious agent, results were consistent (Figure 3).

In summary, shortening the treatment duration for ICU patients with infections is possible and not detrimental for most of them. Indeed, the diversity of patients enrolled in those trials and the consistency of the findings suggest that the conclusions may be applicable to most critically ill patients who develop infections, including severe sepsis or septic shock, with the possible exception of those who are immunosuppressed, those who are infected with multiresistant microorganisms or whose course deteriorates despite treatment, or those whose initial regimen was inappropriate for the responsible pathogens. That strategy should help contain health-care costs and limit in-ICU emergence of bacterial resistance.

## Implementing a structured antibiotic stewardship program

Optimizing in-ICU antimicrobial therapy is difficult. No single measure alone can succeed, emphasizing the need to devise a structured antibiotic stewardship program. Unfortunately, the exact set of key interventions essential to this multifaceted and multidisciplinary ‘care bundle’ remains unknown, as do the factors contributing to its success [1,3,109,144-146]. The interventions should be packaged so that compliance is readily assessable and achievable, which usually means that each bundle includes no more than five to eight interventions. Table 1 provides an example of antibiotic stewardship for patients with VAP. Successful implementation requires an interdisciplinary team, educational interventions, system innovations, process indicator evaluation, and feedback to health-care workers. Several studies using quasi-experimental designs confirmed the usefulness of such a strategy to optimize in-ICU antibiotic stewardship, but not all designs proved to be effective [111,147,148]. As the results of a recent study [149] showed, simply having a reference checklist, without a robust implementation-and-adherence strategy, is unlikely to improve patient outcomes [149,150].

**Table 1:** **A personal care bundle for optimizing antimicrobial treatment for intensive care unit patients with ventilator-associated pneumonia**

**Antibiotic stewardship items**	**Rationale**
Step 1: Obtain bronchoalveolar specimens for Gram staining and cultures before introducing new antibiotics.	Every effort should be made to obtain reliable specimens from the specific infection site for direct microscope examination and cultures in order to enable de-escalation.
Step 2: Start antibiotics less than 2 hours after bronchoalveolar lavage.	Time to appropriate antimicrobial administration is a major outcome determinant for intensive care unit patients with severe bacterial infections.
Step 3: Start therapy using broad-spectrum antibiotics unless no risk factors for resistant pathogens are present.	Owing to the emergence of multiresistant GNB (for example, *Pseudomonas aeruginosa* and ESBL-producing GNB), empirical broad-spectrum antibiotics are justified for most patients with clinically suspected VAP.
Step 4: Stop therapy on day 3 if infection becomes unlikely.	Antibiotics can be discontinued very early when VAP diagnosis becomes highly unlikely based on negative cultures and clinical course and the elimination of an extrapulmonary infection.
Step 5: Use pharmacokinetic-pharmacodynamic data to optimize treatment.	Clinical and bacteriological outcomes can be improved by optimizing the therapeutic regimen according to pharmacokinetic-pharmacodynamic properties of the selected agents.
Step 6: Streamline antibiotic therapy by using narrower-spectrum antibiotics once the etiological agent is identified.	For many patients with VAP, including those with late-onset infections, therapy can be narrowed once respiratory tract and blood culture results become available, either because an anticipated bacterium (for example, *P. aeruginosa*, *Acinetobacter* spp., or methicillin-resistant *Staphylococcus aureus*) was not recovered or because the isolated pathogen is sensitive to a narrower-spectrum antibiotic than that used initially.
Step 7: Switch to monotherapy on days 3 to 5.	Using a two-antibiotic regimen for more than 3 to 5 days has no clinical benefits, provided that initial therapy was appropriate, the clinical course evolves favorably, and microbiological data exclude difficult-to-treat microorganisms.
Step 8: Shorten the treatment duration based on procalcitonin kinetics.	Shorter antibiotic administration for patients with VAP has achieved good outcomes with less antibiotic consumption. Prolonged therapy leads to colonization with antibiotic-resistant bacteria, which may precede recurrent VAP episodes.

ESBL, extended-spectrum β-lactamase; GNB, Gram-negative bacilli; VAP, ventilator-associated pneumonia.

Computerized decision-support programs linked to electronic patient records can facilitate the dissemination of information to physicians for immediate use in therapeutic decision making and improving quality of care [151-154]. Partially or non-automated protocols, often instigated by hospital-based quality-improvement teams, also had demonstrated efficacy [154-157]. A prospective intervention of having an infectious disease specialist interact regularly with the medical ICU team was conducted to assess guideline compliance and antibiotic and health-care costs; it achieved significantly reduced use of extended-spectrum penicillins, carbapenems, vancomycin, and metronidazole [157]. Specifically, the intervention group had a significantly lower rate of treatments not corresponding to guidelines, with fewer MV days, shorter stays, and lower in-hospital mortality. Moreover, $89,944 was saved for early antibiotic discontinuation alone [157].

## Conclusions

The high antibiotic resistance observed in ICU patients who develop infections limits treatment options and justifies using regimens combining several broad-spectrum antibiotics, even when the presumed infection probability is low, because initial inappropriate therapy has been linked to poor prognoses. More than its economic impact, this ‘spiraling empirical’ practice increasingly leads to undue antibiotic administration to many ICU patients without true infections, paradoxically causing the emergence of more antibiotic-resistant microorganisms causing infections that, in turn, are associated with heightened mortality and morbidity. Therefore, antibiotic therapy for ICU patients with infections should be viewed as a two-stage process: the first involves administering broad-spectrum antibiotics to avoid inappropriate treatment of true bacterial infections, and the second focuses on trying to achieve the first without antibiotic overuse or abuse. In general, the first goal can be accomplished by rapidly identifying patients with infection and starting empirical therapy likely to treat the institution’s most common etiological agents. This strategy requires that initial antibiotic choices be guided by local antibiotic resistance patterns and laboratory test results (including Gram staining), rapidly yielding identities of likely responsible pathogens. The second aim involves stopping therapy when the probability of infection is low, focusing and narrowing treatment once the microorganism is known, switching to monotherapy after day 3 whenever possible, and shortening treatment to 7 to 8 days for most patients, based on the clinical response and bacteriology findings. Therefore, every effort should be made to obtain reliable specimens from the specific suspected infection site in each patient for direct microscope examination and cultures in order to de-escalate antibiotics.

## Key messages

- The rapid in-ICU emergence and dissemination of multidrug-resistant microorganisms worldwide constitute a problem of crisis dimensions that is linked directly to inappropriate antimicrobial use.
- Appropriate antibiotic stewardship is a two-stage process.
- Stage I includes rapidly identifying patients with infection, starting an empirical regimen likely to treat the institution’s most common etiological agents, and optimizing bacterial killing by adjusting antibiotic doses and administration modalities based on PK-PD characteristics.
- Stage II involves stopping therapy in patients unlikely to have infections, focusing and narrowing treatment once the responsible pathogen is known, switching to monotherapy after day 3 whenever possible, and shortening antibiotic administration to 7 to 8 days for most patients, based on the therapeutic response and microbiology data.
- Any antibiotic stewardship program should be implemented in a structured manner and requires an interdisciplinary team, educational interventions, system innovations, process indicator evaluation, and feedback to health-care workers.

## Note

This article is part of a series on *Antibiotic resistance in the ICU*, edited by Steven Opal. Other articles in this series can be found at http://ccforum.com/series/antibioticresistance.

## Abbreviations

AUC: Area under the concentration curve
CAI: Community-acquired infection
GNB: Gram-negative bacilli
HAI: Hospital-acquired infection
HAP: Hospital-acquired pneumonia
IDSA: Infectious Diseases Society of America
MIC: Minimal inhibitory concentration
MRSA: Methicillin-resistant *Staphylococcus aureus*
MV: Mechanical ventilation
PCT: Procalcitonin
PK-PD: Pharmacokinetic-pharmacodynamic
VAP: Ventilator-associated pneumonia
